# Echocardiography-Guided Management of Preterms With Patent Ductus Arteriosus Influences the Outcome: A Cohort Study

**DOI:** 10.3389/fped.2020.582735

**Published:** 2020-12-21

**Authors:** Gianluca Terrin, Maria Di Chiara, Giovanni Boscarino, Paolo Versacci, Violante Di Donato, Antonella Giancotti, Elisabetta Pacelli, Francesca Faccioli, Elisa Onestà, Chiara Corso, Alessandra Ticchiarelli, Mario De Curtis

**Affiliations:** Department of Maternal and Child Health, University of Rome La Sapienza, Rome, Italy

**Keywords:** newborn, morbidity, intraventricular hemorrhage (IVH), very low birth weight (VLBW), survival

## Abstract

**Introduction:** Echocardiography (ECHO) with color flow Doppler is considered as the gold standard to identify a hemodynamic patent ductus arteriosus (hs-PDA). However, the optimal diagnostic and therapeutic management for newborns with hs-PDA is still controversial. We aimed to investigate two clinical strategies: (1) targeted treatment based on ECHO criteria and (2) treatment based on ECHO criteria in addition to clinical signs and symptoms.

**Materials and Methods:** This is a cohort study including all neonates consecutively admitted in the Neonatal Intensive Care Unit of University La Sapienza in Rome, with gestational age <32 weeks or body birth weight <1,500 g and with a diagnosis of hs-PDA as confirmed by ECHO evaluation performed within 72 h of life. We classified the babies in two cohorts: (A) pharmacological treatment immediately after ECHO screening and (B) pharmacological therapy for PDA was administered when the relevance of a hs-PDA was associated with clinical signs of hemodynamic instability.

**Results:** We considered as primary outcome newborns who survived without any morbidities (A: 48.1% vs. B: 22.2%, *p* = 0.022). In particular, we found that the rate of intraventricular hemorrhage stage ≥2 was increased in cohort B (A: 3.7% vs. B 24.4%, *p* = 0.020). A multivariate analysis showed that assignment to cohort A independently influences the primary outcome.

**Conclusions:** Adopting an hs-PDA management option based on ECHO-directed therapy regardless of symptoms may reduce the morbidity and improve the survival of very low birth weight infants.

## Introduction

Patent ductus arteriosus (PDA) is essential for fetal life (1). Normally, the ductus arteriosus closes within a few days after birth. In preterm infants, the ductus fails to close, leading to a left heart volume overload and systemic hypoperfusion (2). The persistence of a PDA may increase the risk of intraventricular hemorrhage (IVH), necrotizing enterocolitis (NEC), and chronic lung disease (CLD) (1, 3).

There is no consensus regarding the optimal diagnostic approach of PDA. The 2D echocardiography (ECHO) with color flow Doppler is the gold standard to identify hemodynamically significant PDA (hs-PDA) (4, 5). Nevertheless, it remains to be clarified if the assessment of a hs-PDA at the ECHO is enough to establish an indication for a pharmacological therapeutic intervention or if symptoms should guide the decision to start therapy. If hs-PDA defined only by echocardiographic criteria may not produce significant clinical effects, in many cases, symptoms appear to manifest when the effects of hemodynamic instability already occurred. Pharmacological intervention (i.e., ibuprofen, indomethacin, paracetamol), the first therapeutic option to close PDA in preterm newborns, is associated with the occurrence of severe side effects. Furthermore, clinical evidence demonstrating a reduction in morbidity and mortality in newborns treated for PDA is still scarce (2, 6–9). These findings, associated with spontaneous ductal closure in a number of preterm newborns, suggest an expectant management of PDA based on clinical observation without pharmacological therapy (10, 11). However, prospective studies reported a significantly higher rate of morbidities when a permissive tolerance of PDA strategy was adopted (12). All these considerations raised controversy regarding whom to treat and when to treat preterm infants with hs-PDA. If some infants might benefit from pharmacological therapy, the criteria for PDA treatment remain currently unclear. The American Academy of Pediatrics and Experts on PDA agree that further studies are advocated to evaluate the efficacy of different diagnostic and therapeutic management of newborns with PDA (13–16). Starting from these considerations, we aimed to compare, in a cohort study, two management options: (1) targeted treatment based on ECHO criteria and (2) treatment based on ECHO criteria in addition to clinical signs and symptoms.

## Materials and Methods

### Study Population

All newborns consecutively admitted to the Neonatal Intensive Care Unit (NICU) of University La Sapienza of Rome from January 2016 to November 2019, with gestational age (GA) <32 weeks or body birth weight (BW) <1,500 g and with a diagnosis of hs-PDA as confirmed by echocardiographic evaluation performed within 72 h of life, were included. Infants with congenital malformations, inborn errors of metabolism, congenital infections, and hospital discharge or death within 72 h of life were excluded (17). A consensus definition for a clinical diagnosis of PDA is lacking (18). We performed a differential diagnosis process in order to exclude conditions that may have a clinical presentation that overlaps those of symptomatic hs-PDA, such as sepsis, pulmonary hemorrhage, and pneumothorax.

We defined PDA as hemodynamically significant in the presence of at least one of the following criteria: (1) PDA ≥1.5 mm, (2) unrestrictive pulsatile transductal flow, (3) left atrial-to-aortic root ratio ≥1.5, and (4) absent (end-) diastolic flow in descending aorta.

We classified the babies in two cohorts. Cohort A included newborns consecutively observed in the first 22 months of the study. After that period, the protocol regarding the management of newborns with hs-PDA was changed; thus, we enrolled newborns in cohort B.

In the first period of the study (cohort A), we started pharmacological treatment immediately after echocardiographic evaluation, while in the second period of the study (cohort B) the newborns received pharmacological therapy for PDA when the relevance of a hs-PDA, at echocardiographic evaluation, was associated with clinical signs of hemodynamic instability including acidosis, increased refill capillary time (evaluated three times consecutively), increased oxygen requirement, oliguria, and persistent hypotension (evaluated within 12 h).

### Outcomes

We considered as primary outcome the rate of newborns who survived without any morbidities upon discharge from NICU. Morbidity was defined as the presence of at least one of the following conditions occurring after enrollment: NEC Bell stage ≥2, IVH stage ≥2 (observed after 72 h of life), retinopathy of prematurity stage ≥2, bronchopulmonary dysplasia (BPD), periventricular leukomalacia, prolonged mechanical ventilation (more than 7 days consecutively), and acute renal failure (1, 2, 14, 15, 19, 20). We considered as secondary outcome the rate of newborns with ductal closure within the first 2 weeks of life.

### Data Collection

Medical staff who were blinded to the study aims evaluated the eligibility of newborns observed in NICU. Physicians who were not involved in the care of the newborns collected the informed consent and all data for statistical analysis. A third-party observer unaware of the study aims collected data on primary and secondary outcomes. A statistician blinded to the study performed data analysis.

Prenatal, perinatal, and postnatal data were prospectively recorded in separate data forms. All infants were monitored until discharge, transfer to other hospital, or death. Data GA, BW, gender, type of delivery, antenatal steroid administration, congenital disorders, and occurrence of all relevant obstetric information were collected (21–23). Apgar score at 5 min after birth, pH on cord blood, respiratory distress syndrome, occurrence of hypotension, feeding tolerance, and other gastrointestinal disorders were also prospectively recorded (22, 24, 25). Data on survival, morbidity, and duration of hospital stay were collected in a separate and codified data form. Diagnosis of the main neonatal morbidities was performed according to the standard criteria by physicians unaware of the study design and aims as previously described (26, 27). Nutritional management was performed as previously described (24, 26, 28). Hypotension was defined as any mean blood pressure value below the infant's postmenstrual age (29).

### Therapeutic Protocol of Newborns With hs-PDA

The first-line therapy for all enrolled newborns was pharmacological treatment. Pharmacological treatment with ibuprofen was the first therapeutic choice for PDA treatment. We administered, as first cycle treatment, a dosing regimen of 10 mg/kg ibuprofen, followed by 5 mg/kg ibuprofen at 24 and 48 h later. In case of contraindications to ibuprofen, we administered a paracetamol dose of 20 mg/kg as first dose, followed by 7.5 mg/kg paracetamol every 6 h thereafter, for a total of 6 days. A second pharmacological cycle was performed in the occurrence of symptoms related to hs-PDA for the two study cohorts. We administered a therapeutic cycle of ibuprofen (10 mg/kg, followed by 5 mg/kg ibuprofen at 24 and 48 h later) or, in case of contraindications to it, a cycle of paracetamol (7.5 mg/kg, every 6 h thereafter, for a total of 6 days).

A third pharmacological cycle was performed still in newborns that showed a slight but not significant improvement of clinical conditions and/or ECHO findings. If clinical conditions remained unchanged or worsened after two pharmacological cycles of treatment, the newborns underwent surgery. After the third pharmacological cycle of treatment, if we observed a further improvement of clinical conditions and/or ECHO findings, we administered a fourth pharmacological cycle with indomethacin for a total of 5 days. In case of reopening of hs-PDA associated with signs of hemodynamic instability, we administered one more cycle of ibuprofen or, if there are contraindications to it, paracetamol at the same dosing regimen before the surgery.

### Cranial Ultrasonography Examination

The transducer frequency was set at 7.5 MHz. Images were recorded in coronal and sagittal planes, according to standard procedure, as previously described (30). The anterior fontanel represented the acoustic window for the optimal visualization of the supratentorial structures, whereas the mastoid fontanel was used to evaluate the cerebellum. The cranial ultrasound (cUS) scans were performed in all preterm infants within the first 72 h (T0) at 7 days of life (T1) and at 28 days of life (T2) contemporarily by two physicians with high training in cUS (more than 200 cUS per year) and who were unaware of the study aims. The two sonographers performed each measure three times, and then they calculated the mean value and reported the results in a specific data form. Each measurement was confirmed after an agreement between the sonographers.

### Ethics

The study was conducted in accordance with the World Medical Association Declaration of Helsinki for medical research involving human subjects. To conduct this research, we compared two historical cohorts of newborns using data collected during a study previously approved by the Ethical Committee (no. 5089). Data are available from the Department of Maternal and Child Health Policlinico Umberto I Hospital, La Sapienza University of Rome, Italy, Institutional Data Access for researchers who meet the criteria for access to confidential data.

### Statistics

Statistical analysis was performed per protocol using Statistical Package for Social Science software (SPSS Inc, Chicago, IL, USA), version 22.0. We assessed statistical significance by the tests described below, considering that the level of significance for all statistical tests was two-sided with *p* < 0.05. We checked for normality using the Shapiro–Wilk test. The mean and 95% confidence interval summarized the continuous variables. We compared the cohorts using χ^2^ test for categorical variables and *t*-test or Mann–Whitney for paired and unpaired variables.

We performed binary logistic regression analysis to evaluate whether gender (female or male), BW in model 1 (>1,500 or ≤ 1,500 g) or GA in model 2 (<29 or ≥29 weeks of PMA), prenatal steroid use (no or yes), alteration of Doppler velocimetry of uterine arteries (no or yes), respiratory distress syndrome (no or yes), and cohort assignment (A or B) influenced primary outcome.

## Results

The number of newborns that met the inclusion criteria was 106. We excluded 34 newborns because of major congenital malformations or inborn errors of metabolism or congenital infections (*n* = 11), death before 72 h of life (*n* = 13), and transfer to another hospital before 72 h of life (*n* = 10). Thus, the analysis of data was performed on 27 and 45 newborns enrolled in cohort A and cohort B, respectively. The baseline clinical characteristics of enrolled newborns were not different in the two study cohorts, as reported in Table 1. In Table 2, we reported the echocardiographic pattern of PDA observed in the two cohorts of the study.

**Table 1:** Baseline clinical characteristics of the study cohorts.

	**Cohort A *n* = 27**	**Cohort B *n* = 45**	***p***
Female	11 (40.7)	22 (48.9)	0.502
Gestational age, median weeks (IQR)	28 (4)	27 (2)	0.135
Birth weight, median g (IQR)	1,020 (500)	900 (480)	0.161
Twins	9 (33.3)	8 (17.8)	0.132
Cesarean section	23 (85.2)	35 (77.8)	0.214
Antenatal steroids^a^	16 (59.2)	27 (60)	0.586
Pregnancy-induced hypertension	8 (29.6)	12 (26.7)	0.444
Alteration of doppler velocimetry of uterine arteries	4 (14.8)	6 (13.3)	0.441
pH at birth, median (IQR)	7.25 (0.17)	7.26 (0.09)	0.187
Respiratory distress syndrome	21 (77.8)	39 (86.7)	0.254
FEF, median days (IQR)	21 (22)	25 (19)	0.926
Hypotension requiring inotropes	4 (14.8)	20 (44.4)	0.009
Temperature at 1st h of life, median °C (IQR)	36.1 (0.7)	36.1 (0.3)	0.289
Normoglycemia	8 (53.3)	15 (40.5)	0.400
APGAR score at 5 min of life, median (IQR)	7 (1)	7 (2)	0.815
Sepsis	6 (22.2)	10 (22.2)	0.620

a *Intramuscular steroid cycle in two doses of 12 mg over a 24-h period. Data are expressed as number (%) when not specified*.

*FEF, full enteral feeding*.

**Table 2:** Echocardiographic pattern of patent ductus arteriosus at the time of diagnosis in the two study cohorts.

	**Cohort A *n* = 27**	**Cohort B *n* = 45**	***p***
Diameter ≥1.5 mm	21 (77.8)	33 (73.3)	0.673
Unrestrictive ductal flow (unidirectional left–right or pulsatile bidirectional)	26 (96.3)	44 (97.8)	0.634
Left atrium/aorta ratio ≥1.5	11 (40.7)	17 (37.8)	0.707
Absent or reverse flow in descending aorta	4 (14.8)	5 (11.1)	0.430

*The diagnosis of hemodynamic significance of ductus arteriosus based on echocardiographic parameters. Data are expressed as number (%)*.

**Figure 1:**
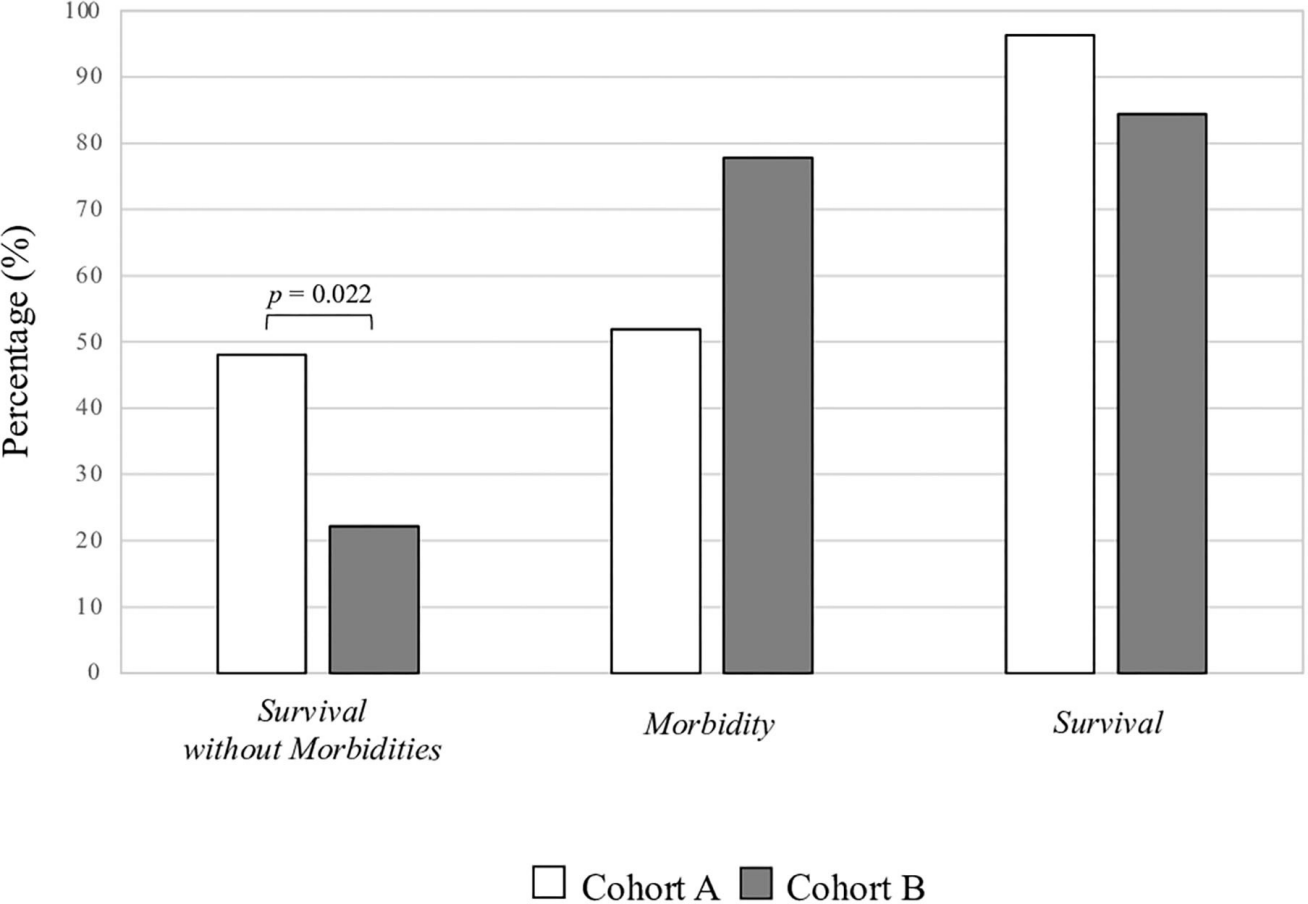
Percentage of morbidity and survival in the two study Cohorts.

The primary outcome (A: 48.1% vs. B: 22.2%, *p* = 0.022) of the study is reported in Figure 1. Among the morbidities observed in the study cohorts, the occurrence of IVH was higher in cohort B compared with that in cohort A (Table 3). The preterm newborns without hs-PDA, of the same study periods, were similar in baseline characteristics and outcomes (Supplementary Table 1). The mean length of hospitalization of patients in cohort A (96.5 days, 95% CI 69.7–123.4) was similar to those in cohort B (78.6 days, 95% CI 64.5–92.6, *p* = 0.185).

**Table 3:** Morbidity in the two study cohorts.

	**Cohort A *n* = 27**	**Cohort B *n* = 45**	***p***
BPD	4 (14.8)	7 (15.6)	0.607
NEC	0 (0)	0 (0)	N/A
IVH	4 (14.8)	13 (28.9)	0.173
Stage ≥2	1 (3.7)	11 (24.4)	0.020
ROP	10 (37)	18 (40)	0.803
Stage ≥ 2	8 (29.6)	9 (20)	0.352
PLV	5 (18.5)	6 (13.3)	0.393
Prolonged invasive mechanical ventilation	4 (14.8)	14 (31.1)	0.122
Acute renal failure	0 (0)	2 (4.4)	0.387

*BPD, bronchopulmonary dysplasia; NEC, necrotizing enterocolitis (Bell stage ≥2); IVH, intraventricular hemorrhage; ROP, retinopathy of prematurity; PLV, periventricular leukomalacia*.

*Data are expressed as number (%)*.

In Table 4, no difference in the rate of ductal closure, reopening, and need of surgical intervention was observed between the study cohorts. The rate of PDA closure before the first 2 weeks of life was similar in cohort A (44.4%) compared with that in cohort B (51.1%, *p* = 0.621). The mean age of definitive ductal closure was similar in cohort A (17.3 days, 95% CI 6.5–28.2) compared with that in cohort B (15.5 days, 95% CI 9.4–21.6, *p* = 0.746).

**Table 4:** Clinical evolution of patent ductus arteriosus.

	**Cohort A *n* = 27**	**Cohort B *n* = 45**	***p***
Close after the first cycle of therapy	21 (77.8)	31 (68.9)	0.596
Definitive closure of PDA	23 (85.2)	39 (86.7)	0.561
Reopening of PDA	5 (18.5)	8 (17.8)	0.576
Surgical intervention	2 (7.4)	1 (2.2)	0.314

*PDA, patency of the ductus arteriosus*.

*Data are expressed as number (%)*.

In Table 5, we report the modalities of treatment in the two cohorts of the study. The rate of newborns treated with specific drugs for PDA was similar between the two cohorts (A: 100% vs. B: 95.6%, *p* = 0.387).

**Table 5:** Pharmacological treatment in the two study cohorts.

	**Cohort A *n* = 27**	**Cohort B *n* = 45**	***p***
Need more than one cycle of therapy, number (%)	4 (14.8)	13 (28.9)	0.143
Number of cycles	1.3 (1.1–1.5)	1.5 (1.2–1.7)	0.277
Start of the first cycle of medical therapy, days	3.8 (2.7–4.9)	3.8 (3.0–4.5)	0.895
Duration of medical therapy, days	4.4 (3.4–5.3)	5.3 (4.3–6.3)	0.202
Paracetamol in the first cycle of medical therapy, number (%)	5 (18.5)	18 (40.0)	0.068
Ibuprofen in the first cycle of medical therapy, number (%)	22 (81.5)	25 (55.6)	0.068
No treatment, number (%)	0 (0.0)	2 (4.4)	0.068

*Data are expressed as mean (95% CI) when not specified*.

A multivariate analysis showed that cohort A assignment independently reduces the risk of combined morbidity and mortality in our population (Table 6).

**Table 6:** Binary logistic regression analysis to evaluate the influence of covariates on primary outcome.

**Primary Outcome**	***B***	***p*-value**	**OR**	**95.0% confidence interval**	
				**Lower limit**	**Upper limit**
**Model 1**					
Birth weight	2.027	0.175	7.588	0.405	142.239
Twins	−0.497	0.519	0.608	0.134	2.758
Pregnancy-induced hypertension	−0.789	0.328	0.454	0.093	2.212
pH on cord blood	−1.176	0.239	0.309	0.044	2.185
Alteration of doppler velocimetry of uterine arteries	−0.149	0.877	0.862	0.130	5.699
Gender	−1.056	0.179	0.348	0.075	1.622
Antenatal corticosteroids^a^	0.324	0.661	1.383	0.324	5.896
Respiratory distress syndrome	0.868	0.336	2.383	0.406	14.000
Cohort	−1.825	0.024	0.161	0.033	0.784
**Model 2**					
Gestational age	−3.913	0.007	0.020	0.001	0.347
Twins	−1.276	0.155	0.279	0.048	1.621
Pregnancy-induced hypertension	−0.892	0.313	0.410	0.073	2.314
pH on cord blood	−1.297	0.258	0.273	0.029	2.603
Alteration of doppler velocimetry of uterine arteries	1.068	0.333	2.909	0.334	25.314
Gender	−1.579	0.083	0.206	0.035	1.227
Antenatal corticosteroids^a^	0.499	0.553	1.648	0.316	8.587
Respiratory distress syndrome	1.526	0.143	4.598	0.596	35.488
Cohort	−2.243	0.020	0.106	0.016	0.703

a *Intramuscular steroid cycle in two doses of 12 mg over a 24-h period*.

## Discussion

We demonstrated that an ECHO-guided therapy regardless of symptoms may increase the probability of survival without morbidity in very low birth weight infants. The reduction in morbidity observed in cohort A was associated with a significant reduction in IVH. Current evidence suggests that many neonatal-related outcomes may depend on the management of PDA. Nevertheless, the literature review reports contradicting findings that mainly rely on the type of the designed study (31). Randomized controlled trials (RCTs) exploring the effects of a prophylactic treatment of PDA found that the occurrence of both death and long-term neurodevelopmental outcomes was not reduced. However, observational studies demonstrated the association between PDA exposure duration and long-term morbidities (14, 32, 33). In addition, retrospective studies found a relationship between symptomatic hs-PDA and morbidity combined with mortality (3).

A Cochrane review from 2010 demonstrated a significant reduction in morbidity associated with prophylactic administration of COX inhibitors in newborns with PDA (9). In contrast, a more recent Cochrane review showed that prophylactic treatment did not result in a reduction of morbidity (8). The concern about the possible exposure of newborns without hs-PDA to unnecessary treatments and the potential harm associated with ductal closure in subjects with elevated pulmonary pressure has raised doubts about the opportunity of the use of medical therapy for hs-PDA (19). In addition, recent evidence demonstrating an unchanged neonatal outcome after the implementation of a more conservative management plan lends support to the argument that PDA treatment should not be useful (6).

A recent retrospective study demonstrated that the rate of spontaneous closure before hospital discharge occurs in the majority of patients (33). However, the authors excluded the deceased infants from the analysis. Indeed out of 26 infants who died, 16 had a recorded cause of death that could be potentially related to PDA, including IVH, and 10 infants who died before 7 days of age had point-of-care ECHO done, and all of them had documented PDA. The authors concluded that the outcome of some deceased infants could have been influenced by the criteria used for treatment (33). Besides that, Okulu et al. in an observational, prospective, and multi-center study, compared the effects of conservative approach and early medical treatment options in newborns with GA <29 weeks. It was found that early treatment was associated with higher mortality and BPD/death. However, in this cohort study, each center used their individual preferences in the decision of PDA management (34).

Thus, it is crucial to identify subjects on whom the advantages of medical treatments outweigh the risks of severe related side effects. To date, evidence regarding when to treat a hs-PDA in a preterm neonate is contrasting. Recent RCTs demonstrated that an early treatment, before the occurrence of symptoms, is safe and results in a lower incidence of late neonatal morbidity (35–37). The DETECT trial on early asymptomatic treatment showed a significant decrease in pulmonary hemorrhage rate in infants treated within the 1st h of life (35). In the PDA-TOLERATE trial enrolling <28-week newborns with a moderate-to-large PDA, the infants were randomized to receive either routine pharmacologic treatment or a conservative approach. The authors did not find any significant differences in terms of PDA closure and late neonatal morbidities. However, they showed that routine treatment, starting from 7 days of life, in infants with GA >26 weeks delayed full enteral feeding and increased the risk of morbidity and death. In this trial, the infants were not enrolled until the end of the 1st week, resulting in 14% of excluded newborns with PDA-related symptoms and 18% of infants not recruited owing to the physician's lack of equipoise (36). Liebowitz et al. starting from these findings, designed an observational study comparing newborns enrolled in the PDA-TOLERATE trial with those infants screened for PDA-TOLERATE who were excluded from the trial (even though they were potentially eligible) because the medical team wanted to use pharmacologic treatment. Interestingly, infants treated earlier within the 1st week of life had a significantly lower incidence of late neonatal morbidity and mortality despite their relative immaturity and their greater need of respiratory support (37).

Our results suggest that an ECHO-guided approach of a PDA, regardless of symptoms, might help to select newborns that should be treated in order to improve their clinical outcomes, hindering a potentially useless drug administration. Targeted treatment based on ECHO parameters may allow for the selection of preterm infants at a high risk of complications prior to the duct becoming clinically significant.

Our results are consistent with those of Rozé et al. This is a French national, prospective, population-based cohort study enrolling extremely preterm infants with GA <29 weeks, who were placed into two groups based on whether they had undergone early ECHO screening or not. The authors reported that an early ECHO-guided treatment was associated with reduction in-hospital mortality and a reduced likelihood of pulmonary hemorrhage, even though they found no differences in late neonatal morbidity (38).

It has been demonstrated that hemodynamic instability is a major risk factor of several late neonatal complications such as IVH, BPD, and NEC (1, 4, 15, 20, 39). Although the accuracy of hs-PDA diagnosis increases with the presence of suggestive symptoms associated with ECHO findings, we hypothesize that waiting for the onset of symptoms may prolong the exposure to hemodynamic instability that, in turn, may increase the rate of morbidity and death. Accordingly, we found an increased morbidity when we decided to treat PDA only after the symptoms' appearance.

The differences in morbidity observed in our study rely mainly on IVH. A retrospective study reported an increased incidence of IVH in infants with persistent exposure to PDA (40). Besides that, it has been shown that medical prophylaxis within the 1st h of life results in a reduction in the incidence of IVH (41).

The literature review on the relation between PDA and IVH is controversial. A recent retrospective cohort study including infants born <29 weeks demonstrated a relation between the presence of hs-PDA and severe IVH. In particular, it was found that a hs-PDA requiring treatment was associated with a higher risk of IVH and that early treatment of hs-PDA was associated with a decreased severity of IVH (40). However, many of previous trials have included infants with PDA regardless of hemodynamic significance (42–44). Therefore, any beneficial effect of a protocol for PDA management may have been masked by the absence of clinical consequences of a physiologic PDA. In addition, the presence of confounders may have influenced the results of these studies. Kim et al. designed a cohort study including newborns with GA <29 weeks to assess the relation between the size of PDA and cerebral hemodynamics within the 1st week of life (45). They indicated that cerebral hemodynamics was found to be stable regardless of the size of PDA in those premature infants. However, the authors did not report a specified protocol for ECHO, which was done at the direction of the attending neonatologist. Moreover, ECHO evaluation was not focused on the hemodynamic significance of the PDA. The strategy adopted in newborns in cohort A has produced a positive effect on the occurrence of IVH, probably by reducing the time of hemodynamic instability. Finally, we speculate that our selective approach may have reduced the possible consequences of hemodynamic instability, due to prolonged exposure to hs-PDA, on cerebral flow. Further studies are needed to establish the causality between the hemodynamic consequences of PDA and the occurrence of IVH.

Our findings should be taking into account several study limitations. The association between early ECHO evaluation and combined morbidity–mortality outcome may be related to the effects of chance (random error), bias, or confounding factors. The two cohorts were similar for conditions affecting morbidity and mortality. We verified that the effect on the main outcome of the study persisted even after correction for confounding variables. In keeping with the available evidence, we included antenatal and postnatal risk factors associated with IVH. Despite of everything, other confounding variables, unknown or not considered in our statistical analysis, may have influenced the study results. This is a small but not a randomized trial. To limit selection bias, physicians prescribing therapy for PDA were not blinded to the ECHO findings and the clinical condition of the baby. This made the study more feasible and affordable but may have compromised its external validity. However, physicians evaluating eligibility were blinded to the study aims and used objective inclusion criteria such as GA and BW. On the other hand, designing a double-blind clinical trial could have influenced the parents' consent of enrollment due to the increasingly strong association between significant PDA and important morbidities. A recent paper reported that the risk of lack of equipoise within physicians caring for infants with hs-PDA could be very high. Hence, it is not easy to design an RCT in newborns of critically ill conditions. The results of this study may encourage designing a specific RCT that could best address this topic. Researchers not involved in eligibility assessment and clinical practice, who were unaware of the cohort assignment to limit observer bias, collected the data for statistical analysis. The protocol for the collection, measurement, and interpretation of data was discussed and defined before starting the study. A statistician blinded to the study performed the analysis. We divided the two study cohorts on a temporal basis. The classification of the subjects on a temporal basis may represent a bias. However, the baseline features were similar between the study cohorts. We did not change the policies during the study period, but we cannot exclude the fact that unknown differences in clinical practice or changes in the medical staff composition may have influenced the outcomes of the study. We used previously described criteria for the definition of hs-PDA (1, 5, 19, 32, 46). Even if other parameters could be useful to define the hemodynamic significance of a PDA, information regarding ECHO findings on pulmonary overflow and systemic steal was not collected. The ECHO data did not include more detailed parameters that could be useful for assessing the hemodynamic significance of a PDA. The ECHO findings collected in this study were limited to the definition of the hemodynamic significance of a PDA. Several of the criteria of hs-PDA are subject to considerable operator variability. Thus, different diagnostic criteria could lead to different conclusions. Our study was focused on short-term outcomes. Further research should investigate the relationship of different strategies to manage PDA closure on brain function and long-term neurodevelopmental outcomes. The small number of the newborns enrolled in the study and the differences in the number of subjects enrolled in the two cohorts limit the generalizability of the results.

In conclusion, an ECHO-based management of PDA regardless of symptoms is a safe and feasible approach useful to improve combined morbidity and mortality outcome in preterm newborns. The optimal protocol for the assessment and treatment of a newborn with hs-PDA should aim not only to an increased rate of PDA closure but also at a reduction in morbidity and mortality. Our results outline the importance of the early identification of newborns with hs-PDA trough ECHO-based protocol and may facilitate the design of a randomized trial on PDA management based on ECHO markers of PDA significance.

## Data Availability Statement

The raw data supporting the conclusions of this article will be made available by the authors, without undue reservation.

## Ethics Statement

Ethical review and approval was not required for the study on human participants in accordance with the local legislation and institutional requirements. Written informed consent to participate in this study was provided by the participants' legal guardian/next of kin.

## Author Contributions

GT, MDi, GB, PV, and EP conceptualized and designed the study, organized the acquisition of data, reviewed, and revised both the analyses and the manuscript. GT, MDi, GB, PV, VDD, AG, EP, FF, EO, CC, AT, and MDe drafted the article and revised it critically for important intellectual content. All the authors approved the final manuscript to be published.

## Conflict of Interest

The authors declare that the research was conducted in the absence of any commercial or financial relationships that could be construed as a potential conflict of interest.
