# Supplementary material for: Echocardiography-Guided Management of Preterms With Patent Ductus Arteriosus Influences the Outcome: A Cohort Study
Source: Front Pediatr. 2020 Dec 21;8:582735. doi: 10.3389/fped.2020.582735 (PMC7779760; doi:10.3389/fped.2020.582735)
Supplement: Supplementary file 1 [file Table_1.DOCX]

**Supplementary Table 1.** Clinical characteristics and outcomes of VLBW without hs-PDA.

|  | **Cohort A**  *n=80* | **Cohort B**  *n=111* | ***p*** |
| --- | --- | --- | --- |
| Female | 35 (43.8) | 51 (45.9) | 0.763 |
| Gestational age, *mean weeks* *(SD)* | 30.2 (2.6) | 29.9 (2.2) | 0.347 |
| Birth weight, *mean g (SD)* | 1296.4 (342.5) | 1316.0 (337.2) | 0.694 |
| Caesarean section | 70 (87.5) | 98 (89.1) | 0.735 |
| Antenatal steroids ^a^ | 55 (68.8) | 85 (76.6) | 0.228 |
| pH at birth, *meann (SD)* | 7.3 (0.1) | 7.3 (0.1) | 0.496 |
| FEF, *mean days (SD)* | 11.3 (9.8) | 11.8 (9.3) | 0.720 |
| Apgar score at 5 minutes of life, *mean (SD)* | 8 (1) | 8 (1) | 0.088 |
| Sepsis | 2 (2.5) | 7 (6.3) | 0.192 |
| BPD | 4 (5) | 3 (2.7) | 0.324 |
| NEC | 1 (1.3) | 4 (3.6) | 0.302 |
| IVH | 2 (2.5) | 5 (4.5) | 0.377 |
| *stage ≥ 2* | 2 (2.5) | 3 (2.7) | 0.653 |
| ROP | 11 (13.8) | 8 (7.2) | 0.136 |
| *stage ≥ 2* | 4 (5) | 2 (1.8) | 0.203 |
| PLV | 1 (1.3) | 3 (2.7) | 0.442 |
| Prolonged invasive mechanical ventilation | 8 (10) | 4 (3.6) | 0.072 |
| Acute renal failure | 0 | 0 | N/A |
| At least one morbidity | 17 (21.3) | 19 (17.1) | 0.471 |
| Exitus | 3 (3.8) | 4 (3.6) | 0.623 |
| Survival without morbidity | 69 (86.3) | 94 (84.7) | 0.763 |

**Notes. (**a) Intramuscular steroid cycle in two doses of 12 mg over a 24-hour period. FEF (Full enteral feeding); BPD (Bronchopulmonary Dysplasia); NEC (Necrotizing Enterocolitis) Bell stage ≥ 2; IVH (Intraventricular Hemorrhage); ROP (Retinopathy of Prematurity); PLV (Periventricular Leukomalacia). Data were expressed as No. (%), when not specified.
